# Magnetic resonance imaging provides evidence of glymphatic drainage from human brain to cervical lymph nodes

**DOI:** 10.1038/s41598-018-25666-4

**Published:** 2018-05-08

**Authors:** Per Kristian Eide, Svein Are Sirirud Vatnehol, Kyrre Eeg Emblem, Geir Ringstad

**Affiliations:** 1 Department of Neurosurgery, Oslo University Hospital-Rikshospitalet, Oslo, Norway; 2 Institute of Clinical Medicine, Faculty of Medicine, University of Oslo, Oslo, Norway; 3 The Intervention Centre, Oslo University Hospital, Oslo, Norway; 4 Department of Diagnostic Physics, Oslo University Hospital, Oslo, Norway; 5 Department of Radiology and Nuclear Medicine, Oslo University Hospital - Rikshospitalet, Oslo, Norway

## Abstract

Pre-clinical research in rodents provides evidence that the central nervous system (CNS) has functional lymphatic vessels. *In-vivo* observations in humans, however, are not demonstrated. We here show data on CNS lymphatic drainage to cervical lymph nodes *in-vivo* by magnetic resonance imaging (MRI) enhanced with an intrathecal contrast agent as a cerebrospinal fluid (CSF) tracer. Standardized MRI of the intracranial compartment and the neck were acquired before and up to 24–48 hours following intrathecal contrast agent administration in 19 individuals. Contrast enhancement was radiologically confirmed by signal changes in CSF nearby inferior frontal gyrus, brain parenchyma of inferior frontal gyrus, parahippocampal gyrus, thalamus and pons, and parenchyma of cervical lymph node, and with sagittal sinus and neck muscle serving as reference tissue for cranial and neck MRI acquisitions, respectively. Time series of changes in signal intensity shows that contrast enhancement within CSF precedes glymphatic enhancement and peaks at 4–6 hours following intrathecal injection. Cervical lymph node enhancement coincides in time with peak glymphatic enhancement, with peak after 24 hours. Our findings provide *in-vivo* evidence of CSF tracer drainage to cervical lymph nodes in humans. The time course of lymph node enhancement coincided with brain glymphatic enhancement rather than with CSF enhancement.

## Introduction

In 2015, the traditional view of the brain having no lymphatic vessels was challenged by evidence showing functional lymphatic vessels lining the cranial dural sinuses in rodents^1,2^. This observation may have profound impact on our understanding of inflammatory and degenerative central nervous system (CNS) diseases. The authors suggested that their findings of lymphatic vessels may represent the second step in the drainage of the interstitial fluid from the brain parenchyma into deep cervical lymph nodes after first been drained into the cerebrospinal fluid (CSF) through a glial “lymphatic” (glymphatic) paravascular pathway. The latter is suggested as a brain-wide route for clearance of water and waste solutes from the brain^3^. Moreover, several reports indicate that reduced glymphatic function may be instrumental in conditions such as Alzheimer’s disease^3^, and post-traumatic illness^4^. Clearance of brain waste metabolites by the glymphatic system is enhanced during sleep^5^.

Our knowledge of CNS lymphatic circulation is largely based on animal studies. Whether differences between species are a determining factor is not known. A recent study has demonstrated presence of meningeal lymphatic vessels in man after administration of intravenous contrast agent at MRI^6^. However, *in-vivo* imaging studies of CNS lymphatic drainage in humans utilizing a tracer substance administered to the CSF have not yet been reported. Human CSF tracer studies might provide better insight into lymphatic drainage of macromolecules from the CNS, and could be pivotal to assess the impact of lymphatic failure in neuroinflammatory and neurodegenerative diseases.

Brain-wide glymphatic circulation was first visualized by MRI after intracisternal MRI contrast agent administration in rats^7^. In a recent cohort study, we have shown the existence of glymphatic parenchymal contrast enhancement in man by applying intrathecal contrast enhanced MRI^8^. Whether the glymphatic system drains to cervical lymph nodes in man remains to be determined.

The present study provides novel *in-vivo* observations of CNS lymphatic drainage in man and demonstrates that CSF tracer uptake in neck lymph nodes is a much slower process than previously observed in animals, and that peak CSF tracer uptake in the glymphatic system and cervical lymph nodes coincides in time.

## Results

**Figure 1:**
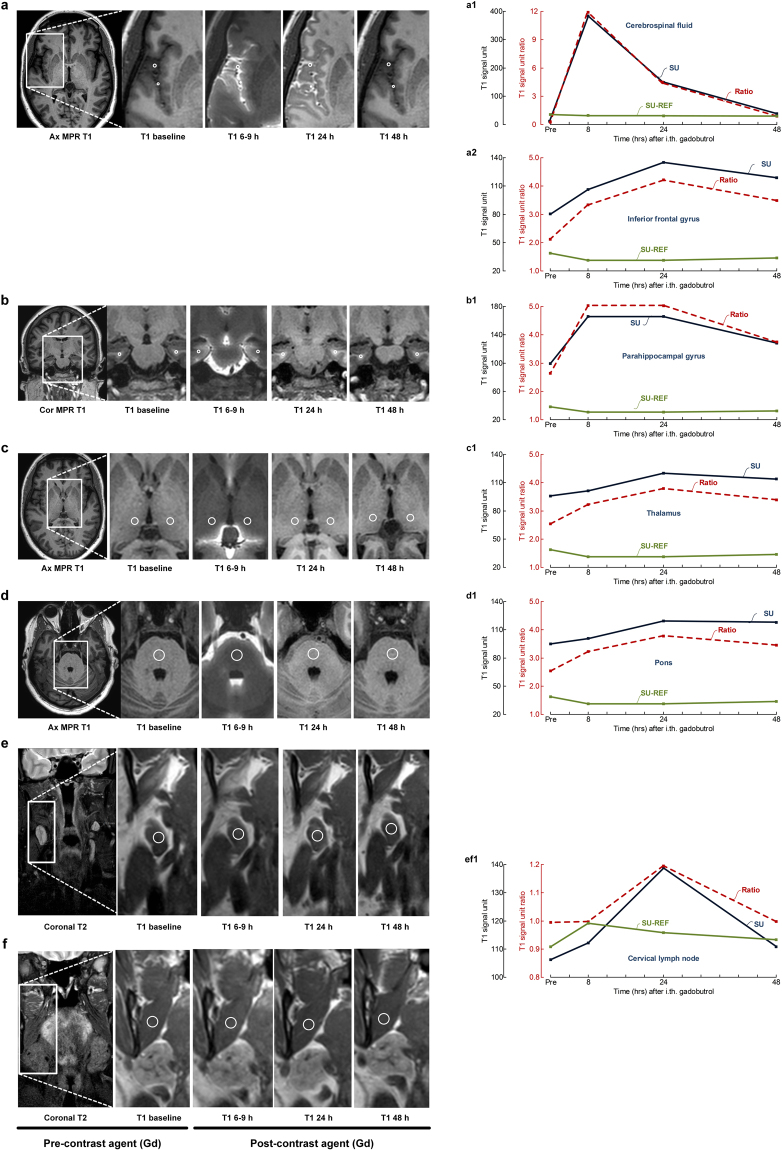
The enrichment of CSF tracer is illustrated in one of the study patients (no 5) at multiple time points within different anatomical regions, including (**a**) CSF nearby inferior frontal gyrus and parenchyma of inferior frontal gyrus, (**b**) parenchyma of parahippocampal gyrus, (**c**) thalamus, (**d**) pons, (**e**) a cervical lymph node. The medial pterygoid muscle (**f**) served as reference tissue in the neck region. The reference tissue from the head region (superior sagittal sinus) is not shown. The left column shows axial (ax) and coronal (Cor) multiplanar reformatting (MPR) T1 images from the head region (**a**–**d**) and coronal neck T2 images (**e**,**f**) to give an overview of the regions from which the magnified images in column 2–5 are retrieved. Column 2 from left presents MRI at baseline (before intrathecal tracer administration; pre) and three subsequent imaging time points (MRI columns 3–5). All images are T1 weighted, except from the image to the left in figure part 1e-f, which is T2 weighted with fat suppression used for location of lymph nodes. The CSF tracer uptake was measured in the T1 weighted images, and in all locations a-f. All measured signal units were normalized against a reference tissue to correct for any baseline shift of image greyscale between time points. The regions of interest are marked by an open circle. Reference tissue was blood of superior sagittal sinus for intracranial MRI (not shown) and medial pterygoid muscle for neck MRI. To the right is presented trend plots of signal units (blue lines for location and green for reference tissue) and signal unit ratios (red stippled lines) for the different anatomical locations in patient no 5, including (a1) CSF nearby inferior frontal gyrus, (a2) parenchyma of inferior frontal gyrus, (b1) parenchyma of parahippocampal gyrus, (c1) parenchyma of thalamus, (d1) parenchyma of pons, and (ef1) parenchyma of cervical lymph node and the medial pterygoid muscle. In this patient, the CSF enrichment nearby inferior frontal gyrus reached maximum after 8 hours (a1), while it reached maximum after 24 hours within inferior frontal gyrus (a2), thalamus (c1), pons (d1) and cervical lymph node (ef1). The CSF tracer enrichment was at the same level after 8 and 24 hours within the parahippocampal gyrus.

This cohort includes 19 individuals who underwent MRI of the head (intracranial) and neck regions with utilization of the contrast agent gadobutrol as CSF tracer (Table 1), and in whom a cervical lymph node with largest diameter >1.5 cm was identified. Figure 1 illustrates from one study individual (no 5) the locations for measurements of MRI signal units in CSF nearby inferior frontal gyrus and parenchyma of inferior frontal gyrus (Fig. 1a), parenchyma of parahippocampal gyrus (Fig. 1b), parenchyma of thalamus (Fig. 1c), parenchyma of pons (Fig. 1d), parenchyma of cervical lymph node (Fig. 1e) and tissue of the medial pterygoid muscle (Fig. 1f) at different time points after intrathecal CSF tracer (gadobutrol) injection. The images are focused on the anatomical areas of study. Figure 1 also presents the trend plots of changes in signal units with the specific regions of interest and within the reference tissue, as well as trends of signal unit ratios.

**Table 1:** Demographic data of patients.

Patient	Age (yrs)	Gender (F/M)	Diagnosis
1	64	M	Idiopathic normal pressure hydrocephalus
2	22	F	Idiopathic intracranial hypotension
3	76	M	Idiopathic normal pressure hydrocephalus
4	25	F	Hydrocephalus
5	47	F	Idiopathic intracranial hypertension
6	28	F	Idiopathic intracranial hypertension
7	33	F	Idiopathic intracranial hypertension
8	34	F	Idiopathic intracranial hypertension
9	39	F	Idiopathic intracranial hypertension
10	19	F	Idiopathic intracranial hypertension
11	71	F	Idiopathic normal pressure hydrocephalus
12	47	F	Arachnoid cyst
13	54	F	Idiopathic intracranial hypertension
14	28	F	Arachnoid cyst
15	54	F	Ventricular cyst
16	20	F	Idiopathic intracranial hypertension
17	45	M	Arachnoid cyst
18	44	M	Idiopathic intracranial hypotension
19	38	F	Arachnoid cyst

F. Female, M: Male.

Table 2 presents the percentage change of signal unit ratios within the CSF and cervical lymph nodes over time after intrathecal gadobutrol administration in the 19 individuals who were included. It should be noted that in two individuals (nos 11 and 13), there was no positive change in signal unit ratio in cervical lymph nodes following intrathecal gadobutrol, and in additional two individuals (nos 12 and 14) only 1% change in signal unit ratio was found. Among the remaining 15 individuals, the signal unit ratio within the lymph node increased maximum 16 ± 8%. In Table 3 is shown the percentage change of signal unit ratios within the brain parenchymal regions of interest (inferior frontal gyrus, parahippocampal gyrus, thalamus and pons) at different time points after intrathecal gadobutrol administration in the 19 individuals. In patient no. 13 there was no positive change in signal unit ratio within intracranial parenchymal regions of interest (Table 3) or the cervical lymph node (Table 2), indicative of no parenchymal CSF tracer enrichment, despite marked increased signal unit ratio within the CSF (Table 2). This observation is extraordinary.

**Table 2:** Percentage change in CSF tracer enrichment within CSF and cervical lymph node at various time points after intrathecal gadobutrol (max increase in bold).

Patient	Time after intrathecal gadobutrol									
	2–4 hours		4–6 hours		6–9 hours		24 hours		48 hours	
	CSF	CLN	CSF	CLN	CSF	CLN	CSF	CLN	CSF	CLN
1	2397	1	**2471**	−1			1611	**5**		
2	3881	2	4919	13	**5138**	5	2104	**26**		
3	2900	−19	**3577**	−9			1793	**15**	128	14
4	72		**1624**	4			955	**16**		
5	5516	−3	**5684**	5	5585	−1	2067	**25**	361	3
6	3929	0	**6478**	2	4943	**8**	3250	1	484	7
7	**1980**	2	1580	−4	1623	−2	336	**12**	119	−7
8	**4431**	1	4125	6			1202	**9**	65	1
9	2881	−5	**3083**	1			1875	**6**		
10	1612	20	1744	**30**	**1764**	24	498	16	115	28
11	3239	−11	4221	−43			**4517**	−9	2343	−14
12	4213	**1**	**4606**	−3	4163	−1	2238	1		
13	507	−8	1198	−7	**1793**	−2	372	−2		
14	412	−6	1687	**1**	**2787**	−2	305	−4	−5	−8
15	3143	−11	**3703**	1	2838	**8**	2817	−1	833	
16	**4246**	12	4246	−2			1226	**19**	207	10
17	1183	**19**	1481	8	**1570**	−1	603	5	121	1
18	1956	4	4402		**4680**	6	1393	**28**	18	1
19	1370	3	1893	0	**1965**	−8	729	−1	−5	**14**
Mean ± STD	2625 ± 1534	0 ± 10	**3301 ± 1585**	0 ± 14	3238 ± 1555	3 ± 8	1573 ± 1110	**9 ± 11**	368 ± 639	4 ± 12

CSF = CSF within Sylvian fissure close to frontal inferior gyrus; CLN = cervical lymph node.

**Table 3:** Percentage change in CSF tracer enrichment within brain parenchyma at various time points (max increase in bold).

Patient	Time after intrathecal gadobutrol																			
	2–4 hours				4–6 hours				6–9 hours				24 hours				48 hours			
	IFG	PHG	THA	PO	IFG	PHG	THA	PO	IFG	PHG	THA	PO	IFG	PHG	THA	PO	IFG	PHG	THA	PO
1	15	25	5	7	10	49	1	−7					**70**	**64**	**11**	**7**				
2	−10	−10	−9	−8	13	28	**23**	11	27	56	9	7	**77**	**81**	16	**23**				
3	7	14	26	15	8	23	17	11					**42**	**52**	**30**	**26**	18	11	4	6
4	9	−1	−3	3	5	5	−7	−9					**90**	**76**	**13**	**18**				
5	19	28	25	20	20	55	22	16	55	**101**	27	26	**96**	101	**47**	**47**	61	45	33	36
6	13	19	16	20	49	71	**49**	**47**	16	54	19	20	**89**	**107**	42	38	48	46	23	33
7	25	27	**23**	21	29	**47**	22	**23**	17	43	6	5	**45**	45	5	3	35	31	11	16
8	9	46	5	7	0	**66**	2	−2					**18**	63	**14**	**21**	10	0	−8	1
9	22	51	21	22	31	106	20	17					**129**	**129**	**43**	**33**				
10	10	17	7	11	17	52	12	8	48	89	5	3	**72**	**65**	**21**	15	40	34	12	**24**
11	23	39	19	15	17	63	2	−1					**122**	**93**	26	18	82	51	**31**	**31**
12	−5	0	−12	−6	13	34	−4	−3	20	45	−6	−6	**63**	**60**	**10**	**11**				
13	−15	−15	−13	−11	−13	−14	−13	−14	−7	−8	−14	−13	−2	−6	−15	−17				
14	−4	−3	−6	−2	−1	−4	−9	−6	10	9	4	4	13	11	−4	−4	**24**	**14**	**10**	**13**
15	−15	−10	−7	−10	−9	4	−10	−7	−14	3	−19	−18	**78**	**72**	**17**	7	46	39	17	**14**
16	35	58	**29**	**27**	28	**114**	16	11					**53**	70	23	12	31	28	11	12
17	−4	14	11	1	8	25	16	12	21	50	18	**14**	**52**	**77**	**27**	9	24	25	11	10
18	−4	4	−3	5	2	16	5	9	−3	14	−4	1	**45**	**67**	**23**	**18**	15	23	7	10
19	6	7	4	7	10	25	7	5	21	35	14	12	**62**	**52**	**20**	**14**	20	19	9	11
Mean ± STD	7 ± 14	16 ± 21	7 ± 14	8 ± 12	13 ± 15	40 ± 35	9 ± 15	6 ± 14	18 ± 20	41 ± 33	5 ± 14	4 ± 13	**64 ± 34**	**67 ± 31**	**19 ± 15**	16 ± 15	35 ± 20	28 ± 15	13 ± 11	**17 ± 11**

IFG = inferior frontal gyrus; PHG = parahippocampal gyrus; THA = thalamus; PO = Pons.

Supplementary Tables 1–6 present the signal units from the locations at study and of the reference tissues, as well as the normalized signal unit ratios for the 19 individuals at the various time points. It should be noted that we lacked MR images at 6–9 hours in 7/19 individuals, which represents a weakness regarding assessment of the time point for peak enhancement.

**Figure 2:**
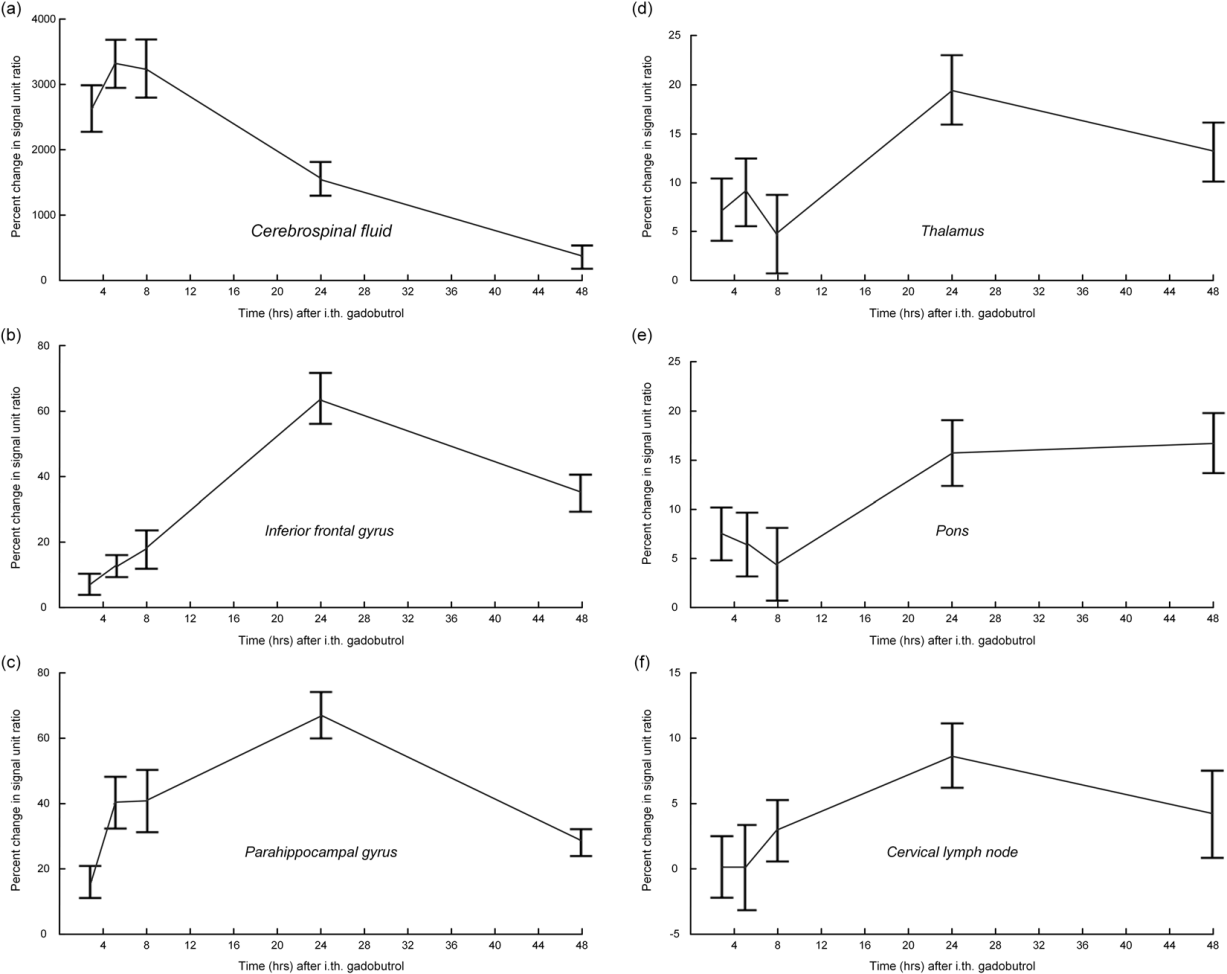
The percentage change in signal unit ratios at different time points after intrathecal CSF tracer (gadobutrol) in the 19 individuals included in the study. Each bar shows the mean ± standard error. The anatomical locations include the (**a**) CSF nearby inferior frontal gyrus, (**b**) parenchyma of inferior frontal gyrus, (**c**) parenchyma of parahippocampal gyrus, (**d**) parenchyma of thalamus, (**e**) parenchyma of pons, and (**f**) parenchyma of a cervical lymph node. While signal unit ratio peaked at 6–9 hours within the CSF (**a**), it peaked at 24 hours within the three brain parenchymal locations (**b**–**e**) and within cervical lymph node (**f**). Linear mixed model analysis revealed significant changes in signal unit ratios over time for all locations: Cerebrospinal fluid (a, P < 0.001), inferior frontal gyrus (b, P < 0.001), parahippocampal gyrus (c, P < 0.001), thalamus (d, P < 0.001), pons (e, P < 0.001), and cervical lymph node (f, P = 0.009).

Among the 19 study individuals, detection of the tracer coincides in time with glymphatic tracer enhancement, where both peaked at 24 hours, while tracer in CSF peaked after 4–6 hours and had declined at 24 hours (Fig. 2a–f). Linear mixed model analysis revealed significant changes in signal unit ratios within all locations (Fig. 2a–f).

Regarding peak enhancement, there was some inter-individual variation. For example, some of the trend plots of individual no. 5 (Fig. 1) differed from trend plots of the entire cohort (Fig. 2). Moreover, peak tracer enhancement occurred in CSF after 4–6 hours in 8/19 individuals (42%) and after 6–9 hours in 7/19 individuals (37%), and after 24–48 hours in 1/19 individuals (5%). In comparison, after 24–48 hours peak enhancement occurred in18/18 (100%) in parenchyma of inferior frontal gyrus, 14/18 individuals (78%) in parahippocampal gyrus, 14/18 individuals (78%) in thalamus and 15/18 individuals (83%) in pons, considering the 18/19 individuals with a positive change in signal unit ratio after intrathecal gadobutrol (Table 4). Within the cervical lymph node, enhancement peaked after 24–48 hours in 11/17 individuals (65%) with a positive change in signal unit ratio after intrathecal gadobutrol. While the timing of peak enhancement within CSF and cervical lymph node differed significantly (P = 0.005; Pearson Chi-square; Table 4), the time point of peak enhancement within brain parenchymal regions and cervical lymph node did not differ significantly (Table 4).

**Table 4:** Time for maximum positive increase in signal unit ratio depending on anatomical region.

Anatomical region	Time after intrathecal gadobutrol					
	2–4 hours	4–6 hours	6–9 hours	24 hours	48 hours	^a^Significance
Cerebrospinal fluid	3	8	7	1	0	=0.005
Inferior frontal gyrus	0	0	0	17	1	=0.10
Parahippocampal gyrus	0	3	1	13	1	=0.58
Thalamus	2	2	0	12	2	=0.65
Pons	1	2	1	10	4	=0.66
Cervical lymph node	2	2	2	10	1	

Data represented by number of individuals within each category (only including individuals with positive change in signal unit ratio at the different time points for each anatomical region). ^a^Significance: Difference with concern to time point of peak enhancement as compared to time point of peak enhancement in cervical lymph node (Pearson Chi-square test).

No serious adverse events were reported following gadobutrol administration.

## Discussion

Our study provides *in-vivo* evidence of lymphatic drainage of a CSF tracer to cervical lymph nodes. Moreover, the time series provide evidence that tracer enhancement within lymph nodes parallels glymphatic enhancement in time.

The study cohort included individuals with various CSF disorders. Therefore, this cohort cannot be considered to consist of healthy individuals, and the present data may not be representative for lymphatic drainage in healthy people. It was, however, beyond the scope of this work to determine how disease type affects lymphatic drainage. In future studies we may utilize the method to compare healthy individuals and patients with various clinical conditions to address how disease affects lymphatic drainage.

Several lines of experimental research in animals have provided evidence of lymphatic drainage from CNS to the cervical lymph nodes along cranial nerves and through the cribriform plate^9–18^. However, the existence of functional, classic lymphatic vessels within the CNS has been disputed. A recent breakthrough was the demonstration of functional dural lymphatic vessels^1,2,6^. It has been proposed that these dural lymphatic vessels may represent the anatomical continuation of the glymphatic system^1^, though it has not been established how the glymphatic system communicates with meningeal lymphatic vessels. It has previously also been suggested that interstitial solutes are drained from the glymphatic system into CSF before resorption into meningeal lymphatics^19^. The present observations, demonstrating that lymph node enhancement coincides with glymphatic, rather than CSF enhancement, favors resorption into lymphatic pathways directly from the glymphatic perivenous space. We therefore hypothesize that the perivenous compartments of the brain and lymphatic vessels are interconnected rather than that subarachnoid CSF and substances drain directly to the meningeal lymphatic pathways.

It should be noted, however, that several aspects of glymphatic circulation are still controversial, including whether interstitial transport is propagated by convective flow or diffusion^20,21^. Drainage to meningeal lymphatic vessels directly from perivenous spaces, as may be inferred by our data, would strongly support a convective force through the interstitial space with direction towards the perivenous compartment.

The assumption that lymphatic drainage to cervical lymph nodes primarily originates from perivascular (glymphatic) transport receives some support from previous experimental animal research showing lymphatic drainage from cerebral perivascular circulation^16,22^, even though these early studies could not define the anatomical link between the perivascular compartment and lymphatic pathways.

While the available evidence for CNS lymphatic drainage to cervical lymph nodes originates from animal research; corresponding *in-vivo* methods for humans have yet not been developed. In a recent cohort study^8^, we reported brain-wide distribution of the contrast agent, and suggested that glymphatic MRI (gMRI) may be utilized to assess glymphatic circulation in man, inspired by previous observations in rodents^7^. The previous human study showed comparable time course for contrast enhancement within several brain regions, with peak after 24 hours. The present data extend and confirm previous observations of parenchymal CSF tracer enrichment within the brain regions studied. In the previous study^8^, we reported that parenchymal contrast enrichment depended on presence of contrast agent in nearby CSF and vicinity to larger, extra-parenchymal arteries. Therefore, the present observation of no parenchymal CSF tracer enrichment in one individual with idiopathic intracranial hypertension (no 13) despite marked enrichment within CSF is extraordinary.

In the present study, the MRI contrast agent gadobutrol was utilized as CSF tracer and detected in cervical lymph nodes in 19 patients. It should be noted that a relative MRI signal increase in tissue at interest, even though when induced by presence of contrast agent cannot be used to quantify contrast agent *concentrations* at this stage. We estimated contrast enhancement in the largest, deep cervical lymph node available for measurement and with a length diameter of at least 15 mm. A threshold of 15 mm was selected to avoid partial volume effects and provide robust measurements. We aimed at exploring contrast enrichment in cervical lymph nodes on a phenomenological basis, since smaller lymph nodes are more susceptible to measurement error.

By normalization of MRI signal unit measurements to standardized reference tissue (superior sagittal sinus and neck muscle, respectively), a change in signal unit can be detected. However, we also measured a signal unit decline in some lymph nodes at a few time points (Table 2), which is most likely due to variations in the measured signal related to stability of the measuring method. While the medial pterygoid muscle should be considered to serve as a robust reference tissue, the muscle is not always in the nearest vicinity of the lymph node at interest, and local magnetic field inhomogeneities might affect these structures differently. Other contributors to measurement error in lymph nodes as well as reference tissue might be image noise and partial averaging effects, even though we strived to minimize these as far as possible by allowing for robust measurements at the central part of the lymph nodes. Nevertheless, despite these sources of error, the tendency for peak glymphatic and lymph node enhancement to coincide still seems clear.

Similar to the venous drainage of the brain, lymphatic drainage to cervical lymph nodes may also show heterogeneity and inter-individual variations. To date, there are no human studies on which particular cervical lymph nodes that receive lymphatic drainage from the intracranial compartment. Hence, before this is further revealed, demonstration of lymph node enhancement with MRI is challenging, underlined by the non-enhancement of lymph nodes in 4 of 19 patients with lymph nodes >15 mm in this study. Due to this heterogeneous pattern, we selected the neck lymph node with the largest degree of enhancement when more than one neck lymph node was available for measurement. Other imaging modalities, such as positron emission tomography, might demonstrate as more sensitive to assess cervical lymph node uptake of CSF tracer.

Our findings point to a unique feature of human lymphatic drainage from the CNS. Early non-enhancement in neck lymph nodes suggests a more limited role of human CSF drainage through nasal lymphatics and perineural root sleeves than previously assumed based on animal studies^23,24^. Moreover, the role of lymphatic vessels lining the dural sinuses could be more profound than previously thought, given their possible function as a main drainage system of the brain-wide glymphatic pathways^1,2^. In the 15 individuals with lymph node enhancement >1%, enhancement peaked at 24–48 hours in 11/15 individuals (73%). In this human cohort, brain glymphatic enhancement typically occurred after 24 hours. This is considerably slower than what is observed in anesthetized mice and rats, where glymphatic enhancement peaks within 2 hours^7^. Comparably, a previous study injecting contrast and dye to the cisterna magna of rats showed much faster lymphatic drainage to cervical lymph nodes^25^ than in the current human cohort. Of note is that MRI of lymph nodes was not done during the time interval of 6–9 hours in 7/19 individuals. Peak enhancement at this time can therefore not be excluded in these individuals.

We observed peak CSF tracer enhancement in the brain parenchymal regions of interest and cervical lymph nodes after 24 hours, i.e. after one night’s sleep (Fig. 2). While this observation is of interest, we cannot conclude whether peak enhancement after 24 hours is related to sleep since we did not perform MRI between the time points 6–9 hours (late afternoon) and 24 hours after administration of contrast agent (next morning). Another factor is that the patients were lying flat until the 6–9 hours scan, and thereafter were allowed to move freely. Therefore, further studies are needed to clarify the role of sleep in human glymphatic circulation, preferably also including a non-sleep control group. From animal studies, it has previously been suggested that sleep has a profound effect on glymphatic circulation and thereby brain clearance of interstitial solutes. Hence, Xie *et al*.^5^ showed that natural sleep or anesthesia is accompanied with a 60% increase in the interstitial space. Furthermore, in that study, the increase in interstitial space increased glymphatic clearance of Aβ twofold. On the other hand, others^26^ using contrast-enhanced MRI and near-infrared fluorescence imaging found that general anesthesia reduced glymphatic function in mice, as compared with awake mice.

Lymphatic failure might be involved both in neuro-immunological and neuro-degenerative diseases. The cerebral lymphatic drainage system may be a pathway for immune cells^10^. In addition, it may be crucial for clearance of toxic waste solutes such as amyloid-β and hyperphosphorylated tau tangles; of which pathologic accumulation in the brain is a hallmark of Alzheimer’s disease^27^. Further development of imaging tools to assess lymphatic clearance may therefore show useful.

In our study, we applied the macrocyclic MRI contrast agent gadobutrol as a CSF tracer. Gadobutrol is well suited for this purpose because of its low molecular size (MW 604 Da). It is also highly hydrophilic and non-ionic, and distributes easily in water^28^. In a previous study assessing glymphatic function in rat brain, the linear MRI contrast agent Gd-DTPA (molecular weight 938 Da) was used^7^. For use in humans, the macrocyclic contrast agents may be preferable above linear agents^29^, because they appear more stable in biological tissue^30^.

Moreover, in previous studies in mice^3,31^, the contrast agent was injected into the cisterna magna at rates and volumes that potentially could overwhelm the intracranial compartment and interfere with CSF circulation^32^. In our study, we injected a total of less than 5 ml fluid into the subarachnoid space at level of the lumbar spine, which should not affect intracranial pressure and CSF flow in humans. Also, there were no serious adverse events following gadobutrol administration.

Comparing the different brain parenchymal regions of interest showed the most pronounced CSF enrichment within the inferior frontal gyrus and the parahippocampal gyrus, which are located close to the leptomeningeal arterial trunks. This observation compares with the view that pulsations are the force behind parenchymal convective glymphatic flow^8,33^.

Assessment of cervical lymph node enhancement might benefit from MRI sequences with image slice of less thickness than applied in this study (3 mm), preferably isotropic 3D volume acquisitions, which also would allow for reconstruction in multiple planes.

A possible cause of cervical lymph node enhancement might be circulating contrast agent in the blood. For example, altered blood-brain-barrier might cause leakage of gadobutrol to the vascular circulation. This seemed, however, less likely since enhancement in reference regions (sagittal sinus for cranial MRI and muscle for neck MRI) was none.

The importance of lymphatic drainage compared to other potential routes for clearance of brain water and solutes is currently not known. Using phantom devices with known concentrations of a given contrast agent in conjunction with MRI acquisitions, it should be possible, however, to quantify lymphatic drainage. An MRI based quantification routine could thereby determine lymphatic failure as a pathogenic factor behind brain disease.

In summary, our study provides *in-vivo* evidence of CSF tracer drainage to cervical lymph nodes in humans. The temporal course of lymph node enhancement coincided in time with brain glymphatic enhancement rather than with CSF enhancement. This is in contrast to previous animal studies demonstrating lymphatic drainage from CSF via the cribriform plate and nasal mucosa, and at a much shorter time span. In the end, improved knowledge of human brain lymphatic drainage is expected to shed new light on pathogenic mechanisms behind degenerative and inflammatory brain diseases.

## Methods

### Ethical permissions

The Institutional Review Board (2015/1868), Regional Ethics Committee (2015/96) and the National Medicines Agency (15/04932-7) approved the study. The study protocol was in accordance with relevant guidelines and regulations. Patients were included after written and oral informed consent.

### Patients

This prospective, observational study included consecutive individuals referred to the Department of neurosurgery, Oslo University Hospital - Rikshospitalet, Oslo, Norway, for various cerebrospinal fluid (CSF) circulation disorders (Table 1).

Exclusion criteria were: History of hypersensitivity reactions to contrast media agents, history of severe allergy reactions in general, evidence of renal dysfunction (i.e. normal glomerular filtration rate, GFR), age <18 or >80 years, pregnant or breastfeeding women.

Inclusion criterion was the identification of a deep cervical lymph node with size >1.5 cm in the cranio-caudal direction. The reason for only including nodes >1.5 cm was to limit partial volume averaging effects from surrounding tissue, mainly from fat, when measuring signal units in lymph nodes.

Among 44 individuals undergoing both head and neck MRI, 19 individuals (43%) had a cervical lymph node >1.5 cm. Hence, the present study cohort includes 19 individuals meeting the inclusion criterion.

This study cohort did not include patients previously reported on.

### MRI protocol

We obtained a series of T1-weighted (w) MRI scans of the intracranial compartment and neck region before and after intrathecal lumbar administration of the MRI contrast agent gadobutrol (0.5 ml of 1.0 mmol/ml; Gadovist^®^, Bayer Pharma AG, GE).

All MRI scans were acquired on a 3 Tesla Philips Ingenia MRI scanner (Philips Medical systems, Best, The Netherlands), using a dedicated imaging protocol for each region, and at all time points. The parameters for 3D T1 w imaging of the intracranial compartment were as follows: repetition time (TR) = 5.1 ms (set to minimum), echo time (TE) = 2.3 ms (set to minimum), flip angle = 8 degrees, field of view = 256 × 256 cm, and matrix = 256 × 256 pixels (reconstructed 512 × 512). We sampled 184 over-contiguous slices with 1 mm thickness, which were automatically reconstructed to 368 slices and a thickness of 0.5 mm. Each image acquisition lasted 6 minutes and 29 seconds. An automated anatomy recognition protocol based on landmark detection in MRI data (SmartExam^TM^, Philips Medical Systems, Best, The Netherlands) was applied at every time point to secure consistency and reproducibility of the MRI studies. Images of the neck were all obtained in an anatomical standardized coronal plane, using T1 w turbo spin echo (TSE) DIXON with main image sequence parameters as follows: TR = 560 ms, TE = 14 ms, flip angle = 90 degrees, field of view 250 × 198 mm, voxel size = 1 × 1 × 3 mm reconstructed to 0.58 × 0.58 × 3 mm^3^, gap 0.3 mm, number of slices = 30. To detect neck lymph nodes, we obtained coronal T2 w TSE DIXON with TR = ranged 2500–3500 (actual 2500), TE = 80 ms, flip angle = 90 degrees, field of view 250 × 200 mm, resolution 0.6 × 0.79 × 3 mm^3^ reconstructed to 0.58 × 0.58 × 3 mm^3^, gap 0.3 mm, number of slices = 30. To ensure same position on the coronal slices between scan times a screen dump showing the placement of the first coronal images was saved and used as a reference for subsequent planning by the radiographer. In general, the center slice was placed at the anterior superior part of the 4^th^ cervical vertebra.

### Intrathecal administration of gadobutrol

The study participants met about 8 a.m. for a pre contrast MR image acquisition and were thereafter transported on a mobile MRI-tabletop to an adjacent operating room, where an interventional neuroradiologist performed x-ray guided lumbar puncture. Intrathecal injection of 0.5 ml of 1.0 mmol/ml gadobutrol (Gadovist^tm^, Bayer Pharma AG, Berlin, Germany) was preceded by verifying correct position of the syringe tip in the subarachnoid space in terms of CSF backflow from the puncture needle, and by injecting a small amount (typically 3 ml) of 270 mg I/ml iodixanol (Visipaque^TM^, GE Healthcare, USA) to confirm unrestricted distribution of radiopaque contrast agent in the lumbar subarachnoid space. Following needle removal, the study subjects were instructed to rotate themselves once around the long axis of the body once before transportation back to the MRI suite, while remaining in the supine position on the same MRI-tabletop.

### Post contrast MRI acquisitions

The study participants were instructed to remain supine in bed during the first MRI cans. All transfer of study subjects between the neurosurgical department and the MRI suite, and between the bed and the MRI table, was performed by the hospital staff to allow for the patient remaining in the supine position. For practical purposes, patients were allowed to move without restrictions after the final MRI of day 1 and until the next morning (approximately 24 hours after contrast agent injection). Thirteen of 19 patients were also scanned at 48 hours.

All the MRI exams were categorized into the following time intervals: Pre-contrast, 2–4 hours, 4–6 hours, 6–9 hours, 24 hours, and 48 hours.

### Image analysis

For each exam, a board-certified neuroradiologist (G.R.) with 11 years’ experience placed circular regions of interest bilaterally and symmetrically as discussed below directly on axially reconstructed T1 weighted images using the hospital picture archiving and communication system (PACS) (Sectra IDS7^®^, Sectra, Sweden). Each region of interest provides the mean signal intensity from the image greyscale, and was normalized to reference tissue to compare values between time points. All regions of interest were fitted to local anatomical landmarks to avoid partial volume effects from neighboring tissue or CSF.

For each exam, the following four regions of interest were used: 1) CSF nearby inferior frontal gyrus (average of left and right sides). 2) Parenchyma of inferior frontal gyrus (average of left and right sides). 3) Parenchyma of parahippocampal gyrus (average of left and right sides). 4) Parenchyma of thalamus (average of left and right sides). 5) Parenchyma of pons (one region of interest). 6) Blood of sagittal sinus within a predefined region above the venous confluence served as reference for measurements in brain parenchyma. 7) Parenchyma of deep cervical lymph node. If a lymph node was identified on the left and right side, we selected the one with largest enhancement following contrast agent, and examined the same lymph node over time. 8) Medial pterygoid muscle as reference tissue for lymph node measurements to correct for any baseline variations in displayed signal intensity values between scans. To limit possible partial volume averaging effects, all ROIs were placed well within the borders of the defined tissue or fluid compartment. Figure 1 illustrates measurements fromdifferent anatomical regions of interest before (pre) and at different time points after gadobutrol administration.

For each time point, we determined the signal unit ratio between the intracranial region of interest and blood of sagittal sinus, and between parenchyma of deep cervical lymph node and medial pterygoid muscle.

### Statistics

Statistical analyses were performed using the SPSS software version 24 (IBM Corporation, Armonk, NY). Statistically significant changes in signal unit ratios over time were determined by linear mixed model analysis. Differences between categorical data were determined using Pearson Chi-square test. Statistical significance was accepted at the 0.05 level (two-tailed).

## Electronic supplementary material

Supplementary Tables 1–6
