# Supplementary material for: Magnetic resonance imaging provides evidence of glymphatic drainage from human brain to cervical lymph nodes
Source: Sci Rep. 2018 May 8;8:7194. doi: 10.1038/s41598-018-25666-4 (PMC5940793; doi:10.1038/s41598-018-25666-4)
Supplement: Supplementary file 1 — Supplementary Tables 1–6 [file 41598_2018_25666_MOESM1_ESM.docx]

**Magnetic resonance imaging provides evidence of glymphatic drainage from human brain to cervical lymph nodes**

Per Kristian Eide^a,b^, Svein Are Sirirud Vatnehol^b,c^, Kyrre Eeg Emblem^c,d^, Geir Ringstad^b,e^

*^a^Dept. of Neurosurgery, Oslo University Hospital-Rikshospitalet, ^b^Institute of Clinical Medicine, Faculty of Medicine, University of Oslo, Oslo, Norway, ^c^The Intervention Centre, Oslo University Hospital, ^d^Dept. of Diagnostic Physics, Oslo University Hospital, ^e^Dept. of Radiology and Nuclear Medicine, Oslo University Hospital - Rikshospitalet,*

**Suppl Table 1. Overview of signal units within cerebrospinal fluid and reference tissue including normalized signal unit ratios, before (Pre) and after intrathecal gadobutrol**

|  | **Pre contrast** | | |  | **Time after intrathecal gadobutrol** | | | | | | | | | | | | | | | | | | |
| --- | --- | --- | --- | --- | --- | --- | --- | --- | --- | --- | --- | --- | --- | --- | --- | --- | --- | --- | --- | --- | --- | --- | --- |
|  |  | | |  | **2 - 4 hours** | | |  | **4-6 hours** | | |  | **6-9 hours** | | |  | **24 hours** | | |  | **48 hours** | | |
| **Patient** | **SU_CSF_** | **SU_REF_** | **Ratio** |  | **SU_CSF_** | **SU_REF_** | **Ratio** |  | **SU_CSF_** | **SU_REF_** | **Ratio** |  | **SU_CSF_** | **SU_REF_** | **Ratio** |  | **SU_CSF_** | **SU_REF_** | **Ratio** |  | **SU_CSF_** | **SU_REF_** | **Ratio** |
| 1 | 14 | 42 | 0.3 |  | 308 | 37 | 8.3 |  | 360 | 42 | 8.6 |  |  |  |  |  | 251 | 44 | 5.7 |  |  |  |  |
| 2 | 6 | 31 | 0.2 |  | 262 | 34 | 7.7 |  | 272 | 28 | 9.7 |  | 294 | 29 | 10.1 |  | 128 | 30 | 4.3 |  |  |  |  |
| 3 | 6 | 45 | 0.1 |  | 156 | 39 | 4.0 |  | 201 | 41 | 4.9 |  |  |  |  |  | 106 | 42 | 2.5 |  | 14 | 46 | 0.3 |
| 4 | 17 | 27 | 0.6 |  | 27 | 25 | 1.1 |  | 293 | 27 | 10.9 |  |  |  |  |  | 186 | 28 | 6.6 |  |  |  |  |
| 5 | 8 | 38 | 0.2 |  | 402 | 34 | 11.8 |  | 414 | 34 | 12.2 |  | 383 | 32 | 12.0 |  | 146 | 32 | 4.6 |  | 33 | 34 | 1.0 |
| 6 | 7 | 42 | 0.2 |  | 235 | 35 | 6.7 |  | 296 | 27 | 11.0 |  | 311 | 37 | 8.4 |  | 201 | 36 | 5.6 |  | 37 | 38 | 1.0 |
| 7 | 20 | 32 | 0.6 |  | 338 | 26 | 13.0 |  | 273 | 26 | 10.5 |  | 323 | 30 | 10.8 |  | 90 | 33 | 2.7 |  | 41 | 30 | 1.4 |
| 8 | 8 | 29 | 0.3 |  | 325 | 26 | 12.5 |  | 338 | 29 | 11.7 |  |  |  |  |  | 97 | 27 | 3.6 |  | 15 | 33 | 0.5 |
| 9 | 12 | 30 | 0.4 |  | 310 | 26 | 11.9 |  | 331 | 26 | 12.7 |  |  |  |  |  | 237 | 30 | 7.9 |  |  |  |  |
| 10 | 22 | 38 | 0.6 |  | 337 | 34 | 9.9 |  | 363 | 34 | 10.7 |  | 367 | 34 | 10.8 |  | 128 | 37 | 3.5 |  | 46 | 37 | 1.2 |
| 11 | 6 | 33 | 0.2 |  | 170 | 28 | 6.1 |  | 275 | 35 | 7.9 |  |  |  |  |  | 277 | 33 | 8.4 |  | 151 | 34 | 4.4 |
| 12 | 8 | 35 | 0.2 |  | 345 | 35 | 9.9 |  | 355 | 33 | 10.8 |  | 341 | 35 | 9.7 |  | 187 | 35 | 5.3 |  |  |  |  |
| 13 | 7 | 38 | 0.2 |  | 47 | 42 | 1.1 |  | 98 | 41 | 2.4 |  | 143 | 41 | 3.5 |  | 40 | 46 | 0.9 |  |  |  |  |
| 14 | 7 | 43 | 0.2 |  | 35 | 42 | 0.8 |  | 128 | 44 | 2.9 |  | 188 | 40 | 4.7 |  | 29 | 44 | 0.7 |  | 6 | 39 | 0.2 |
| 15 | 8 | 28 | 0.3 |  | 278 | 30 | 9.3 |  | 326 | 30 | 10.9 |  | 277 | 33 | 8.4 |  | 250 | 30 | 8.3 |  | 80 | 30 | 2.7 |
| 16 | 22 | 86 | 0.3 |  | 667 | 60 | 11.1 |  | 856 | 77 | 11.1 |  |  |  |  |  | 285 | 84 | 3.4 |  | 69 | 88 | 0.8 |
| 17 | 48 | 152 | 0.3 |  | 559 | 138 | 4.1 |  | 644 | 129 | 5.0 |  | 691 | 131 | 5.3 |  | 291 | 131 | 2.2 |  | 102 | 146 | 0.7 |
| 18 | 15 | 142 | 0.1 |  | 291 | 134 | 2.2 |  | 623 | 131 | 4.8 |  | 712 | 141 | 5.0 |  | 194 | 123 | 1.6 |  | 17 | 136 | 0.1 |
| 19 | 38 | 110 | 0.3 |  | 523 | 103 | 5.1 |  | 709 | 103 | 6.9 |  | 742 | 104 | 7.1 |  | 298 | 104 | 2.9 |  | 37 | 113 | 0.3 |
| **Mean ± STD** | **15 ± 12** | **54 ± 39** | **0.3 ± 0.2** |  | **296 ± 170** | **49 ± 35** | **7 ± 4** |  | **377 ± 197** | **49 ± 34** | **9 ± 3** |  | **398 ± 203** | **57 ± 42** | **8 ± 3** |  | **180 ± 85** | **51 ± 33** | **4 ± 2** |  | **50 ± 41** | **62 ± 43** | **1.1 ± 1.2** |

SU = signal unit; CSF = CSF within Sylvian fissure close to frontal inferior gyrus; REF = blood of superior sagittal sinus; Ratio = signal units within CSF divided by signal units within REF; STD = standard deviation.

**Suppl Table 2. Overview of signal units within parenchyma of inferior frontal gyrus and reference tissue including normalized signal unit ratios, before (Pre) and after intrathecal gadobutrol**

|  | **Pre contrast** | | |  | **Time after intrathecal gadobutrol** | | | | | | | | | | | | | | | | | | |
| --- | --- | --- | --- | --- | --- | --- | --- | --- | --- | --- | --- | --- | --- | --- | --- | --- | --- | --- | --- | --- | --- | --- | --- |
|  |  | | |  | **2 - 4 hours** | | |  | **4-6 hours** | | |  | **6-9 hours** | | |  | **24 hours** | | |  | **48 hours** | | |
| **Patient** | **SU_IFG_** | **SU_REF_** | **Ratio** |  | **SU_IFG_** | **SU_REF_** | **Ratio** |  | **SU_IFG_** | **SU_REF_** | **Ratio** |  | **SU_IFG_** | **SU_REF_** | **Ratio** |  | **SU_IFG_** | **SU_REF_** | **Ratio** |  | **SU_IFG_** | **SU_REF_** | **Ratio** |
| 1 | 81 | 42 | 1.93 |  | 82 | 37 | 2.22 |  | 89 | 42 | 2.12 |  |  |  |  |  | 144 | 44 | 3.27 |  |  |  |  |
| 2 | 80 | 31 | 2.58 |  | 79 | 34 | 2.32 |  | 82 | 28 | 2.93 |  | 95 | 29 | 3.28 |  | 137 | 30 | 4.57 |  |  |  |  |
| 3 | 79 | 45 | 1.76 |  | 73 | 39 | 1.87 |  | 78 | 41 | 1.90 |  |  |  |  |  | 105 | 42 | 2.50 |  | 95 | 46 | 2.07 |
| 4 | 76 | 27 | 2.81 |  | 77 | 25 | 3.08 |  | 80 | 27 | 2.96 |  |  |  |  |  | 150 | 28 | 5.36 |  |  |  |  |
| 5 | 82 | 38 | 2.16 |  | 87 | 34 | 2.56 |  | 88 | 34 | 2.59 |  | 107 | 32 | 3.34 |  | 135 | 32 | 4.22 |  | 118 | 34 | 3.47 |
| 6 | 89 | 42 | 2.12 |  | 84 | 35 | 2.40 |  | 85 | 27 | 3.15 |  | 91 | 37 | 2.46 |  | 144 | 36 | 4.00 |  | 119 | 38 | 3.13 |
| 7 | 86 | 32 | 2.69 |  | 87 | 26 | 3.35 |  | 90 | 26 | 3.46 |  | 94 | 30 | 3.13 |  | 129 | 33 | 3.91 |  | 109 | 30 | 3.63 |
| 8 | 83 | 29 | 2.86 |  | 81 | 26 | 3.12 |  | 83 | 29 | 2.86 |  |  |  |  |  | 91 | 27 | 3.37 |  | 104 | 33 | 3.15 |
| 9 | 75 | 30 | 2.50 |  | 79 | 26 | 3.04 |  | 85 | 26 | 3.27 |  |  |  |  |  | 172 | 30 | 5.73 |  |  |  |  |
| 10 | 86 | 38 | 2.26 |  | 85 | 34 | 2.50 |  | 90 | 34 | 2.65 |  | 114 | 34 | 3.35 |  | 144 | 37 | 3.89 |  | 117 | 37 | 3.16 |
| 11 | 65 | 33 | 1.97 |  | 68 | 28 | 2.43 |  | 81 | 35 | 2.31 |  |  |  |  |  | 144 | 33 | 4.36 |  | 122 | 34 | 3.59 |
| 12 | 82 | 35 | 2.34 |  | 78 | 35 | 2.23 |  | 87 | 33 | 2.64 |  | 98 | 35 | 2.80 |  | 134 | 35 | 3.83 |  |  |  |  |
| 13 | 87 | 38 | 2.29 |  | 82 | 42 | 1.95 |  | 82 | 41 | 2.00 |  | 87 | 41 | 2.12 |  | 103 | 46 | 2.24 |  |  |  |  |
| 14 | 83 | 43 | 1.93 |  | 78 | 42 | 1.86 |  | 84 | 44 | 1.91 |  | 85 | 40 | 2.13 |  | 96 | 44 | 2.18 |  | 93 | 39 | 2.38 |
| 15 | 85 | 28 | 3.04 |  | 77 | 30 | 2.57 |  | 83 | 30 | 2.77 |  | 86 | 33 | 2.61 |  | 162 | 30 | 5.40 |  | 133 | 30 | 4.43 |
| 16 | 221 | 86 | 2.57 |  | 208 | 60 | 3.47 |  | 254 | 77 | 3.30 |  |  |  |  |  | 331 | 84 | 3.94 |  | 296 | 88 | 3.36 |
| 17 | 256 | 152 | 1.68 |  | 223 | 138 | 1.62 |  | 235 | 129 | 1.82 |  | 268 | 131 | 2.05 |  | 335 | 131 | 2.56 |  | 306 | 146 | 2.10 |
| 18 | 257 | 142 | 1.81 |  | 232 | 134 | 1.73 |  | 242 | 131 | 1.85 |  | 248 | 141 | 1.76 |  | 323 | 123 | 2.63 |  | 284 | 136 | 2.09 |
| 19 | 195 | 110 | 1.77 |  | 194 | 103 | 1.88 |  | 200 | 103 | 1.94 |  | 223 | 104 | 2.14 |  | 298 | 104 | 2.87 |  | 241 | 113 | 2.13 |
| **Mean ± STD** | **113 ± 65** | **54 ± 39** | **2.3 ± 0.4** |  | **108 ± 57** | **49 ± 35** | **2.4 ± 0.6** |  | **116 ± 63** | **49 ± 34** | **2.5 ± 0.6** |  | **133 ± 70** | **57 ± 42** | **2.6 ± 0.6** |  | **172 ± 82** | **51 ± 33** | **3.7 ± 1.1** |  | **164 ± 83** | **62 ± 43** | **3.0 ± 0.8** |

SU = signal unit; IFG = brain tissue of inferior frontal gyrus; REF = blood of superior sagittal sinus; Ratio = signal units within IFG divided by signal units within REF; STD = standard deviation.

**Suppl Table 3. Overview of signal units within parenchyma of parahippocampal gyrus and reference tissue including normalized signal unit ratios, before (Pre) and after intrathecal gadobutrol**

|  | **Pre contrast** | | |  | **Time after intrathecal gadobutrol** | | | | | | | | | | | | | | | | | | |
| --- | --- | --- | --- | --- | --- | --- | --- | --- | --- | --- | --- | --- | --- | --- | --- | --- | --- | --- | --- | --- | --- | --- | --- |
|  |  | | |  | **2 - 4 hours** | | |  | **4-6 hours** | | |  | **6-9 hours** | | |  | **24 hours** | | |  | **48 hours** | | |
| **Patient** | **SU_PHG_** | **SU_REF_** | **Ratio** |  | **SU_PHG_** | **SU_REF_** | **Ratio** |  | **SU_PHG_** | **SU_REF_** | **Ratio** |  | **SU_PHG_** | **SU_REF_** | **Ratio** |  | **SU_PHG_** | **SU_REF_** | **Ratio** |  | **SU_PHG_** | **SU_REF_** | **Ratio** |
| 1 | 102 | 42 | 2.43 |  | 112 | 37 | 3.03 |  | 152 | 42 | 3.62 |  |  |  |  |  | 175 | 44 | 3.98 |  |  |  |  |
| 2 | 98 | 31 | 3.16 |  | 97 | 34 | 2.85 |  | 113 | 28 | 4.04 |  | 143 | 29 | 4.93 |  | 172 | 30 | 5.73 |  |  |  |  |
| 3 | 98 | 45 | 2.18 |  | 97 | 39 | 2.49 |  | 110 | 41 | 2.68 |  |  |  |  |  | 139 | 42 | 3.31 |  | 111 | 46 | 2.41 |
| 4 | 104 | 27 | 3.85 |  | 95 | 25 | 3.80 |  | 109 | 27 | 4.04 |  |  |  |  |  | 190 | 28 | 6.79 |  |  |  |  |
| 5 | 98 | 38 | 2.58 |  | 112 | 34 | 3.29 |  | 136 | 34 | 4.00 |  | 166 | 32 | 5.19 |  | 166 | 32 | 5.19 |  | 127 | 34 | 3.74 |
| 6 | 101 | 42 | 2.40 |  | 100 | 35 | 2.86 |  | 111 | 27 | 4.11 |  | 137 | 37 | 3.70 |  | 179 | 36 | 4.97 |  | 133 | 38 | 3.50 |
| 7 | 99 | 32 | 3.09 |  | 102 | 26 | 3.92 |  | 118 | 26 | 4.54 |  | 133 | 30 | 4.43 |  | 148 | 33 | 4.48 |  | 122 | 30 | 4.07 |
| 8 | 100 | 29 | 3.45 |  | 131 | 26 | 5.04 |  | 166 | 29 | 5.72 |  |  |  |  |  | 152 | 27 | 5.63 |  | 114 | 33 | 3.45 |
| 9 | 95 | 30 | 3.17 |  | 124 | 26 | 4.77 |  | 170 | 26 | 6.54 |  |  |  |  |  | 218 | 30 | 7.27 |  |  |  |  |
| 10 | 103 | 38 | 2.71 |  | 108 | 34 | 3.18 |  | 140 | 34 | 4.12 |  | 174 | 34 | 5.12 |  | 165 | 37 | 4.46 |  | 134 | 37 | 3.62 |
| 11 | 91 | 33 | 2.76 |  | 107 | 28 | 3.82 |  | 157 | 35 | 4.49 |  |  |  |  |  | 176 | 33 | 5.33 |  | 142 | 34 | 4.18 |
| 12 | 112 | 35 | 3.20 |  | 112 | 35 | 3.20 |  | 142 | 33 | 4.30 |  | 162 | 35 | 4.63 |  | 179 | 35 | 5.11 |  |  |  |  |
| 13 | 109 | 38 | 2.87 |  | 102 | 42 | 2.43 |  | 101 | 41 | 2.46 |  | 108 | 41 | 2.63 |  | 124 | 46 | 2.70 |  |  |  |  |
| 14 | 106 | 43 | 2.47 |  | 100 | 42 | 2.38 |  | 104 | 44 | 2.36 |  | 107 | 40 | 2.68 |  | 120 | 44 | 2.73 |  | 110 | 39 | 2.82 |
| 15 | 107 | 28 | 3.82 |  | 103 | 30 | 3.43 |  | 119 | 30 | 3.97 |  | 130 | 33 | 3.94 |  | 197 | 30 | 6.57 |  | 159 | 30 | 5.30 |
| 16 | 246 | 86 | 2.86 |  | 272 | 60 | 4.53 |  | 472 | 77 | 6.13 |  |  |  |  |  | 409 | 84 | 4.87 |  | 321 | 88 | 3.65 |
| 17 | 299 | 152 | 1.97 |  | 309 | 138 | 2.24 |  | 318 | 129 | 2.47 |  | 387 | 131 | 2.95 |  | 455 | 131 | 3.47 |  | 359 | 146 | 2.46 |
| 18 | 278 | 142 | 1.96 |  | 274 | 134 | 2.04 |  | 298 | 131 | 2.27 |  | 314 | 141 | 2.23 |  | 401 | 123 | 3.26 |  | 328 | 136 | 2.41 |
| 19 | 232 | 110 | 2.11 |  | 233 | 103 | 2.26 |  | 272 | 103 | 2.64 |  | 296 | 104 | 2.85 |  | 334 | 104 | 3.21 |  | 284 | 113 | 2.51 |
| **Mean ± STD** | **136 ± 69** | **54 ± 39** | **2.8 ± 0.6** |  | **142 ± 71** | **49 ± 35** | **3.2 ± 0.9** |  | **174 ± 98** | **49 ± 34** | **3.9 ±1.3** |  | **188 ± 92** | **57 ± 42** | **3.8 ± 1.1** |  | **216 ± 102** | **51 ± 33** | **4.7 ± 1.4** |  | **188 ± 96** | **62 ± 43** | **3.4 ± 0.9** |

SU = signal unit; PHG = brain tissue of parahippocampal gyrus; REF = blood of superior sagittal sinus; Ratio = signal units within PHG divided by signal units within REF; STD = standard deviation.

**Suppl Table 4. Overview of signal units within thalamus and reference tissue including normalized signal unit ratios, before (Pre) and after intrathecal gadobutrol**

|  | **Pre contrast** | | |  | **Time after intrathecal gadobutrol** | | | | | | | | | | | | | | | | | | |
| --- | --- | --- | --- | --- | --- | --- | --- | --- | --- | --- | --- | --- | --- | --- | --- | --- | --- | --- | --- | --- | --- | --- | --- |
|  |  | | |  | **2 - 4 hours** | | |  | **4-6 hours** | | |  | **6-9 hours** | | |  | **24 hours** | | |  | **48 hours** | | |
| **Patient** | **SU_THA_** | **SU_REF_** | **Ratio** |  | **SU_THA_** | **SU_REF_** | **Ratio** |  | **SU_THA_** | **SU_REF_** | **Ratio** |  | **SU_THA_** | **SU_REF_** | **Ratio** |  | **SU_THA_** | **SU_REF_** | **Ratio** |  | **SU_THA_** | **SU_REF_** | **Ratio** |
| 1 | 118 | 42 | 2.81 |  | 109 | 37 | 2.95 |  | 119 | 42 | 2.83 |  |  |  |  |  | 137 | 44 | 3.11 |  |  |  |  |
| 2 | 103 | 31 | 3.32 |  | 103 | 34 | 3.03 |  | 114 | 28 | 4.07 |  | 105 | 29 | 3.62 |  | 116 | 30 | 3.87 |  |  |  |  |
| 3 | 79 | 45 | 1.76 |  | 86 | 39 | 2.21 |  | 84 | 41 | 2.05 |  |  |  |  |  | 96 | 42 | 2.29 |  | 84 | 46 | 1.83 |
| 4 | 105 | 27 | 3.89 |  | 94 | 25 | 3.76 |  | 98 | 27 | 3.63 |  |  |  |  |  | 123 | 28 | 4.39 |  |  |  |  |
| 5 | 96 | 38 | 2.53 |  | 107 | 34 | 3.15 |  | 105 | 34 | 3.09 |  | 103 | 32 | 3.22 |  | 119 | 32 | 3.72 |  | 114 | 34 | 3.35 |
| 6 | 91 | 42 | 2.17 |  | 88 | 35 | 2.51 |  | 87 | 27 | 3.22 |  | 95 | 37 | 2.57 |  | 111 | 36 | 3.08 |  | 101 | 38 | 2.66 |
| 7 | 102 | 32 | 3.19 |  | 102 | 26 | 3.92 |  | 101 | 26 | 3.88 |  | 101 | 30 | 3.37 |  | 110 | 33 | 3.33 |  | 106 | 30 | 3.53 |
| 8 | 101 | 29 | 3.48 |  | 95 | 26 | 3.65 |  | 103 | 29 | 3.55 |  |  |  |  |  | 107 | 27 | 3.96 |  | 106 | 33 | 3.21 |
| 9 | 82 | 30 | 2.73 |  | 86 | 26 | 3.31 |  | 85 | 26 | 3.27 |  |  |  |  |  | 117 | 30 | 3.90 |  |  |  |  |
| 10 | 100 | 38 | 2.63 |  | 96 | 34 | 2.82 |  | 100 | 34 | 2.94 |  | 94 | 34 | 2.76 |  | 118 | 37 | 3.19 |  | 109 | 37 | 2.95 |
| 11 | 82 | 33 | 2.48 |  | 83 | 28 | 2.96 |  | 89 | 35 | 2.54 |  |  |  |  |  | 103 | 33 | 3.12 |  | 111 | 34 | 3.26 |
| 12 | 109 | 35 | 3.11 |  | 96 | 35 | 2.74 |  | 99 | 33 | 3.00 |  | 102 | 35 | 2.91 |  | 120 | 35 | 3.43 |  |  |  |  |
| 13 | 105 | 38 | 2.76 |  | 101 | 42 | 2.40 |  | 98 | 41 | 2.39 |  | 97 | 41 | 2.37 |  | 108 | 46 | 2.35 |  |  |  |  |
| 14 | 109 | 43 | 2.53 |  | 100 | 42 | 2.38 |  | 101 | 44 | 2.30 |  | 105 | 40 | 2.63 |  | 107 | 44 | 2.43 |  | 109 | 39 | 2.79 |
| 15 | 98 | 28 | 3.50 |  | 98 | 30 | 3.27 |  | 95 | 30 | 3.17 |  | 93 | 33 | 2.82 |  | 123 | 30 | 4.10 |  | 123 | 30 | 4.10 |
| 16 | 246 | 86 | 2.86 |  | 222 | 60 | 3.70 |  | 256 | 77 | 3.32 |  |  |  |  |  | 295 | 84 | 3.51 |  | 279 | 88 | 3.17 |
| 17 | 270 | 152 | 1.78 |  | 271 | 138 | 1.96 |  | 265 | 129 | 2.05 |  | 274 | 131 | 2.09 |  | 296 | 131 | 2.26 |  | 287 | 146 | 1.97 |
| 18 | 280 | 142 | 1.97 |  | 255 | 134 | 1.90 |  | 270 | 131 | 2.06 |  | 266 | 141 | 1.89 |  | 299 | 123 | 2.43 |  | 287 | 136 | 2.11 |
| 19 | 225 | 110 | 2.05 |  | 219 | 103 | 2.13 |  | 226 | 103 | 2.19 |  | 242 | 104 | 2.33 |  | 256 | 104 | 2.46 |  | 251 | 113 | 2.22 |
| **Mean ± STD** | **132 ± 67** | **54 ± 39** | **2.7 ± 0.6** |  | **127 ± 62** | **49 ± 35** | **2.9 ± 0.6** |  | **131 ± 66** | **49 ± 34** | **2.9 ± 0.6** |  | **140 ± 73** | **57 ± 42** | **2.7 ± 0.5** |  | **151 ± 73** | **51 ± 33** | **3.2 ± 0.7** |  | **159 ± 82** | **62 ± 43** | **2.9 ± 0.7** |

SU = signal unit; THA = brain tissue of thalamus; REF = blood of superior sagittal sinus; Ratio = signal units within THA divided by signal units within REF; STD = standard deviation.

**Suppl Table 5. Overview of signal units within pons and reference tissue including normalized signal unit ratios, before (Pre) and after intrathecal gadobutrol**

|  | **Pre contrast** | | |  | **Time after intrathecal gadobutrol** | | | | | | | | | | | | | | | | | | |
| --- | --- | --- | --- | --- | --- | --- | --- | --- | --- | --- | --- | --- | --- | --- | --- | --- | --- | --- | --- | --- | --- | --- | --- |
|  |  | | |  | **2 - 4 hours** | | |  | **4-6 hours** | | |  | **6-9 hours** | | |  | **24 hours** | | |  | **48 hours** | | |
| **Patient** | **SU_PONS_** | **SU_REF_** | **Ratio** |  | **SU_PONS_** | **SU_REF_** | **Ratio** |  | **SU_PONS_** | **SU_REF_** | **Ratio** |  | **SU_PONS_** | **SU_REF_** | **Ratio** |  | **SU_PONS_** | **SU_REF_** | **Ratio** |  | **SU_PONS_** | **SU_REF_** | **Ratio** |
| 1 | 88 | 42 | 2.10 |  | 83 | 37 | 2.24 |  | 82 | 42 | 1.95 |  |  |  |  |  | 99 | 44 | 2.25 |  |  |  |  |
| 2 | 104 | 31 | 3.35 |  | 105 | 34 | 3.09 |  | 104 | 28 | 3.71 |  | 104 | 29 | 3.59 |  | 124 | 30 | 4.13 |  |  |  |  |
| 3 | 87 | 45 | 1.93 |  | 87 | 39 | 2.23 |  | 88 | 41 | 2.15 |  |  |  |  |  | 102 | 42 | 2.43 |  | 94 | 46 | 2.04 |
| 4 | 101 | 27 | 3.74 |  | 96 | 25 | 3.84 |  | 92 | 27 | 3.41 |  |  |  |  |  | 124 | 28 | 4.43 |  |  |  |  |
| 5 | 96 | 38 | 2.53 |  | 103 | 34 | 3.03 |  | 100 | 34 | 2.94 |  | 102 | 32 | 3.19 |  | 119 | 32 | 3.72 |  | 117 | 34 | 3.44 |
| 6 | 94 | 42 | 2.24 |  | 94 | 35 | 2.69 |  | 89 | 27 | 3.30 |  | 99 | 37 | 2.68 |  | 111 | 36 | 3.08 |  | 113 | 38 | 2.97 |
| 7 | 105 | 32 | 3.28 |  | 103 | 26 | 3.96 |  | 105 | 26 | 4.04 |  | 103 | 30 | 3.43 |  | 112 | 33 | 3.39 |  | 114 | 30 | 3.80 |
| 8 | 97 | 29 | 3.34 |  | 93 | 26 | 3.58 |  | 95 | 29 | 3.28 |  |  |  |  |  | 109 | 27 | 4.04 |  | 111 | 33 | 3.36 |
| 9 | 86 | 30 | 2.87 |  | 91 | 26 | 3.50 |  | 87 | 26 | 3.35 |  |  |  |  |  | 114 | 30 | 3.80 |  |  |  |  |
| 10 | 105 | 38 | 2.76 |  | 104 | 34 | 3.06 |  | 101 | 34 | 2.97 |  | 97 | 34 | 2.85 |  | 118 | 37 | 3.19 |  | 127 | 37 | 3.43 |
| 11 | 79 | 33 | 2.39 |  | 77 | 28 | 2.75 |  | 83 | 35 | 2.37 |  |  |  |  |  | 93 | 33 | 2.82 |  | 107 | 34 | 3.15 |
| 12 | 108 | 35 | 3.09 |  | 102 | 35 | 2.91 |  | 99 | 33 | 3.00 |  | 101 | 35 | 2.89 |  | 120 | 35 | 3.43 |  |  |  |  |
| 13 | 110 | 38 | 2.89 |  | 108 | 42 | 2.57 |  | 102 | 41 | 2.49 |  | 103 | 41 | 2.51 |  | 110 | 46 | 2.39 |  |  |  |  |
| 14 | 106 | 43 | 2.47 |  | 101 | 42 | 2.40 |  | 102 | 44 | 2.32 |  | 103 | 40 | 2.58 |  | 104 | 44 | 2.36 |  | 109 | 39 | 2.79 |
| 15 | 102 | 28 | 3.64 |  | 98 | 30 | 3.27 |  | 102 | 30 | 3.40 |  | 98 | 33 | 2.97 |  | 117 | 30 | 3.90 |  | 125 | 30 | 4.17 |
| 16 | 276 | 86 | 3.21 |  | 245 | 60 | 4.08 |  | 274 | 77 | 3.56 |  |  |  |  |  | 303 | 84 | 3.61 |  | 317 | 88 | 3.60 |
| 17 | 286 | 152 | 1.88 |  | 263 | 138 | 1.91 |  | 272 | 129 | 2.11 |  | 280 | 131 | 2.14 |  | 268 | 131 | 2.05 |  | 303 | 146 | 2.08 |
| 18 | 289 | 142 | 2.04 |  | 285 | 134 | 2.13 |  | 291 | 131 | 2.22 |  | 289 | 141 | 2.05 |  | 295 | 123 | 2.40 |  | 304 | 136 | 2.24 |
| 19 | 243 | 110 | 2.21 |  | 244 | 103 | 2.37 |  | 240 | 103 | 2.33 |  | 257 | 104 | 2.47 |  | 263 | 104 | 2.53 |  | 277 | 113 | 2.45 |
| **Mean ± STD** | **135 ± 75** | **54 ± 39** | **2.7 ± 0.6** |  | **131 ± 69** | **49 ± 35** | **2.9 ± 0.6** |  | **132 ± 74** | **49 ± 34** | **2.9 ± 0.6** |  | **145 ± 79** | **57 ± 42** | **2.8 ± 0.5** |  | **148 ± 72** | **51 ± 33** | **3.2 ± 0.7** |  | **171 ± 91** | **62 ± 43** | **3.0 ± 0.7** |

SU = signal unit; REF = blood of superior sagittal sinus; Ratio = signal units within pons divided by signal units within REF; STD = standard deviation.

**Suppl Table 6. Overview of signal units within cervical lymph node and reference tissue including normalized signal unit ratios, before (Pre) and after intrathecal gadobutrol**

|  | **Pre contrast** | | |  | **Time after intrathecal gadobutrol** | | | | | | | | | | | | | | | | | | |
| --- | --- | --- | --- | --- | --- | --- | --- | --- | --- | --- | --- | --- | --- | --- | --- | --- | --- | --- | --- | --- | --- | --- | --- |
|  |  | | |  | **2 - 4 hours** | | |  | **4-6 hours** | | |  | **6-9 hours** | | |  | **24 hours** | | |  | **48 hours** | | |
| **Patient** | **SU_CLN_** | **SU_REF_** | **Ratio** |  | **SU_CLN_** | **SU_REF_** | **Ratio** |  | **SU_CLN_** | **SU_REF_** | **Ratio** |  | **SU_CLN_** | **SU_REF_** | **Ratio** |  | **SU_CLN_** | **SU_REF_** | **Ratio** |  | **SU_CLN_** | **SU_REF_** | **Ratio** |
| 1 | 113 | 105 | 1.08 |  | 111 | 102 | 1.09 |  | 112 | 105 | 1.07 |  |  |  |  |  | 125 | 111 | 1.13 |  |  |  |  |
| 2 | 112 | 125 | 0.90 |  | 102 | 112 | 0.91 |  | 117 | 116 | 1.01 |  | 111 | 118 | 0.94 |  | 135 | 120 | 1.13 |  |  |  |  |
| 3 | 110 | 103 | 1.07 |  | 98 | 113 | 0.87 |  | 103 | 106 | 0.97 |  |  |  |  |  | 125 | 102 | 1.23 |  | 123 | 101 | 1.22 |
| 4 | 120 | 119 | 1.01 |  |  |  |  |  | 127 | 121 | 1.05 |  |  |  |  |  | 141 | 121 | 1.17 |  |  |  |  |
| 5 | 106 | 111 | 0.95 |  | 108 | 116 | 0.93 |  | 110 | 110 | 1.00 |  | 113 | 119 | 0.95 |  | 138 | 116 | 1.19 |  | 111 | 113 | 0.98 |
| 6 | 101 | 110 | 0.92 |  | 99 | 108 | 0.92 |  | 100 | 107 | 0.93 |  | 114 | 115 | 0.99 |  | 105 | 113 | 0.93 |  | 113 | 115 | 0.98 |
| 7 | 124 | 98 | 1.27 |  | 125 | 97 | 1.29 |  | 119 | 98 | 1.21 |  | 122 | 98 | 1.24 |  | 135 | 95 | 1.42 |  | 116 | 99 | 1.17 |
| 8 | 112 | 110 | 1.02 |  | 126 | 123 | 1.02 |  | 131 | 121 | 1.08 |  |  |  |  |  | 125 | 113 | 1.11 |  | 112 | 109 | 1.03 |
| 9 | 121 | 112 | 1.08 |  | 119 | 116 | 1.03 |  | 118 | 108 | 1.09 |  |  |  |  |  | 130 | 113 | 1.15 |  |  |  |  |
| 10 | 124 | 124 | 1.00 |  | 121 | 101 | 1.20 |  | 134 | 103 | 1.30 |  | 130 | 105 | 1.24 |  | 129 | 111 | 1.16 |  | 136 | 106 | 1.28 |
| 11 | 107 | 93 | 1.15 |  | 106 | 103 | 1.03 |  | 73 | 112 | 0.65 |  |  |  |  |  | 106 | 101 | 1.05 |  | 105 | 106 | 0.99 |
| 12 | 129 | 121 | 1.07 |  | 132 | 122 | 1.08 |  | 120 | 116 | 1.03 |  | 125 | 118 | 1.06 |  | 132 | 122 | 1.08 |  |  |  |  |
| 13 | 136 | 122 | 1.11 |  | 129 | 126 | 1.02 |  | 128 | 123 | 1.04 |  | 131 | 120 | 1.09 |  | 142 | 130 | 1.09 |  |  |  |  |
| 14 | 117 | 117 | 1.00 |  | 114 | 121 | 0.94 |  | 118 | 117 | 1.01 |  | 119 | 122 | 0.98 |  | 122 | 127 | 0.96 |  | 118 | 128 | 0.92 |
| 15 | 142 | 106 | 1.34 |  | 127 | 107 | 1.19 |  | 142 | 105 | 1.35 |  | 150 | 104 | 1.44 |  | 143 | 108 | 1.32 |  |  |  |  |
| 16 | 228 | 223 | 1.02 |  | 277 | 241 | 1.15 |  | 233 | 233 | 1.00 |  |  |  |  |  | 305 | 250 | 1.22 |  | 290 | 257 | 1.13 |
| 17 | 277 | 263 | 1.05 |  | 219 | 175 | 1.25 |  | 254 | 223 | 1.14 |  | 230 | 221 | 1.04 |  | 273 | 248 | 1.10 |  | 268 | 252 | 1.06 |
| 18 | 205 | 230 | 0.89 |  | 224 | 241 | 0.93 |  |  |  |  |  | 232 | 245 | 0.95 |  | 274 | 240 | 1.14 |  | 208 | 232 | 0.90 |
| 19 | 209 | 204 | 1.02 |  | 234 | 221 | 1.06 |  | 224 | 218 | 1.03 |  | 223 | 236 | 0.94 |  | 229 | 226 | 1.01 |  | 258 | 221 | 1.17 |
| **Mean ± STD** | **142 ± 50** | **137 ± 51** | **1.05 ± 0.11** |  | **143 ± 55** | **136 ± 49** | **1.05 ± 0.12** |  | **137 ± 49** | **130 ± 44** | **1.05 ± 0.15** |  | **150 ± 48** | **143 ± 55** | **1.07 ± 0.16** |  | **159 ± 61** | **140 ± 54** | **1.14 ± 0.12** |  | **163 ± 71** | **153 ± 65** | **1.07 ± 0.12** |

SU = signal unit; CLN = cervical lymph node; REF = medial pterygoid muscle; Ratio = signal units within CLN divided by signal units within REF; STD = standard deviation.
